# TB-diabetes co-morbidity in Ghana: The importance of *Mycobacterium africanum* infection

**DOI:** 10.1371/journal.pone.0211822

**Published:** 2019-02-07

**Authors:** Adwoa Asante-Poku, Prince Asare, Nyonuku Akosua Baddoo, Audrey Forson, Pius Klevor, Isaac Darko Otchere, Sammy Yaw Aboagye, Stephen Osei-Wusu, Emelia Konadu Danso, Kwadwo Koram, Sebastien Gagneux, Dorothy Yeboah-Manu

**Affiliations:** 1 Noguchi Memorial Institute for Medical Research, College of Health Sciences, University of Ghana, Legon Accra, Ghana; 2 West African Centre for Cell Biology of Infectious Pathogens, University of Ghana, Legon, Accra, Ghana; 3 Department of Chest Diseases, Korle-Bu Teaching Hospital, Korle-Bu, Accra, Ghana; 4 Department of Medical Parasitology and Infection Biology, Swiss Tropical and Public Health Institute, Basel, Switzerland; 5 University of Basel, Basel, Switzerland; The Foundation for Medical Research, INDIA

## Abstract

**Background:**

Diabetes Mellitus (DM) is a known risk factor for tuberculosis (TB) but little is known on TB-Diabetes Mellitus (TBDM) co-morbidity in Sub-Saharan Africa.

**Methods:**

Consecutive TB cases registered at a tertiary facility in Ghana were recruited from September 2012 to April 2016 and screened for DM using random blood glucose and glycated hemoglobin (HbA1c) level. TB patients were tested for other clinical parameters including HIV co-infection and TB lesion location. Mycobacterial isolates obtained from collected sputum samples were characterized by standard methods. Associations between TBDM patients’ epidemiological as well as microbiological variables were assessed.

**Results:**

The prevalence of DM at time of diagnosis among 2990 enrolled TB cases was 9.4% (282/2990). TBDM cases were significantly associated with weight loss, poor appetite, night sweat and fatigue (p<0.001) and were more likely (p<0.001) to have lower lung cavitation 85.8% (242/282) compared to TB Non-Diabetic (TBNDM) patients 3.3% (90/2708). We observed 22.3% (63/282) treatment failures among TBDM patients compared to 3.8% (102/2708) among TBNDM patients (p<0.001). We found no significant difference in the TBDM burden attributed by *M*. *tuberculosis* sensu stricto (Mtbss) and *Mycobacterium africanum* (Maf) and (Mtbss; 176/1836, 9.6% and Maf; 53/468, 11.3%, p = 0.2612). We found that diabetic individuals were suggestively likely to present with TB caused by *M*. *africanum* Lineage 6 as opposed to *Mtbss* (odds ratio (OR) = 1.52; 95% confidence interval (CI): 0.92–2.42, p = 0.072).

**Conclusion:**

Our findings confirms the importance of screening for diabetes during TB diagnosis and highlights the association between genetic diversity and diabetes. in Ghana.

## Introduction

Tuberculosis (TB) remains a major cause of morbidity and mortality worldwide, with an estimated 10.4 million people newly developing TB and 1.7 million dying from it in 2016 [1]. Over 70% of these new cases occurred in developing countries and the highest rate of death occurred in Africa. HIV infection and the emergence of multidrug-resistant (MDR) TB strains which complicates treatments are well recognized threats to control of TB. However, other factors also contribute to the TB burden, including smoking [2] substance abuse [3], vitamin D deficiency [4], and diabetes mellitus (DM) [5, 6]. DM also affects TB disease presentation and treatment response. On the other hand, active TB disease might induce glucose intolerance and worsen glycaemic control in people with DM. Individuals with DM have been reported to have a 3–fold higher risk of developing active TB, compared to those without DM [7]. Moreover, TBDM cases have a higher likelihood of delayed diagnosis, poorer prognosis, and increased severity of symptoms and mortality for both diseases [8].

DM is on the rise; in 2016, the International Diabetes Federation (IDF) estimated a global DM burden of about 422 million cases, projected to hit 642 million cases by 2040 (over 60% increase) [9]. The association between TB and DM has been considered for many years, though most research works was carried out in the developed countries because in the past, non-communicable diseases such as DM have not been considered of public health relevance in developing countries [10]. However, this trend is changing and DM is increasingly becoming a public health importance in developing countries including sub–Saharan Africa. It has been projected that by 2040, more than 28 million DM cases will be in Africa, driven mainly by recent rapid urbanization and changing lifestyles [9, 11]. The rise in DM cases coupled with the relative high rate of TB now makes Africa an important region to study TBDM.

In Africa, studies investigating the prevalence of TB among persons with DM are limited. The few studies that have been performed show varying prevalence of DM in TB patients, ranging from 3% [12] to 36% [13], more than 5 folds greater than in the general population [14,15]. In Tanzania, 1.3% of screened adults with DM had TB, 7–fold greater than the general population [16]. A review of 13 observational studies, of which only one was from a low-income country and none from Africa, found that diabetes was associated with TB regardless of study design [17].

Routine screening of TB patients for DM is lacking in most African health facilities, somewhat because of cost, perceived complexities [18] and non-existent treatment infrastructure. The standard diagnostic methods present their own challenges. Patients are required to fast for several hours before fasting blood glucose (FBG), or the the oral glucose tolerance test (OGTT). In addition, patients are to have specific time-bound measurements and/or interpretations affected by transient hyperglycaemia hence necessitating multiple measurements [19, 20]. Use of random blood glucose (RBG) and glycated haemoglobin (HbA1c) with the latter being more expensive, is sensitive, does not require the patient to fast and can be offered at point of care (POC) to minimize the time to diagnosis [21]. As HbA1c is less affected by transient hyperglycaemia, it also reduces the need for multiple repeat tests.

Ghana is one of the high TB-HIV burden countries in sub-Saharan Africa. The few studies conducted in the country gave a national DM prevalence rate of 4.6% in individuals aged 18–69 years [22]. Nonetheless, no study to date has investigated the association of DM with clinical presentations, treatment outcomes as well as infecting species of the *Mycobacterium tuberculosis* complex (MTBC). Thus, this study was conducted to estimate the prevalence of DM among newly diagnosed TB patients and search for associated clinical and microbiological features.

## Materials and methods

### Ethical statement

The Institutional Review Board (IRB) of the Noguchi Memorial Institute for Medical Research, Legon-Ghana approved all study protocols and written consent format used for this study; Federal wide Assurance number FWA00001824. Informed consent was sought from each case prior to study enrolment. In accordance with ethical review board regulation in Ghana, consent was sought from guardians of children below the ages of 18 years before enrolment into the study and in some cases child assent was also sought.

### Study participant inclusion criteria

This was an observational cross-sectional study in which newly diagnosed sputum smear-positive adult TB cases registered at a tertiary facility in Ghana before the commencement of TB treatment from September 2012 to April 2016 were recruited. To confirm the initial diagnosis at the health facility and to identify the infecting mycobacterial species, sputum specimen was collected from each study participant, following the National Tuberculosis Control Program guidelines. Samples were taken only after a detailed explanation of the study aims and written or thumb-printed consent have been obtained for participation. In addition, clinical characteristics of cases including previous history of TB, HIV, DM as well as patient’s demography and epidemiological data were obtained from each participant. Patient recruited were aged between 3–86 years.

### Diabetes and chest X-ray screening

DM screening was done according to the American Diabetes Association (ADA) criteria. A glucometer (ACCU-CHEK) Active Glucose Monitoring System, Roche Diabetes Care Limited, Burgess Hill, UK was used for DM screening. All eligible patients irrespective of known diagnosis of DM were investigated as follows: Random blood glucose test was first performed by means of the finger prick test using OneTouch Ultra test strips and if the level was less than 7mmol/L, no further action was taken. If the RBG was 7mmol/L or higher, 5ml of venous blood was taken and transported to the laboratory for further assessment by HbA1c evaluation within 4 hours of collection. Patients with HbA1c levels <6% were considered to have normal glycemic level 5.7–6.4% prediabetics, and levels 6.5% and above were confirmed as having DM. All patients were subjected to chest radiography (posterior-anterior view). The pre-treatment chest radiograph was read by two qualified pulmonologists blinded to patients’ disease status. Reading of the chest radiographs focused on lung parenchymal opacity and cavitation. Radiological lesions on chest X-ray were classified into minimal, moderately advanced, and far advanced as per American Thoracic Society (ATS) criteria.

### Categorization of participants

All recruited participants confirmed as having both TB and DM disease status were categorized as TBDM group where as non-diabetic TB participants were categorized as TBNDM.

### Mycobacterial isolation and species characterisation

The sputum samples were processed for culture on solid media and were re-examined for the presence of AFBs. The obtained isolates were then confirmed as a species of the MTBC by detecting IS*6110* as previously described [23]. The main phylogenetic lineages classification was done using TaqMan real-time PCR (TaqMan, Applied Bio systems, USA) probes targeting lineage-specific SNPs as reported by Stucki *et al* while [24] sub-lineage were defined using spoligotyping [25]. Patterns obtained were interpreted according to SITVITWEB database [26] (http://www.pasteur-guadeloupe.fr: 8081/SITVIT_ONLINE). SITVITWEB assigned shared types numbers were used whenever a spoligotyping pattern was found in the database while families and subfamilies were assigned based on the MIRU-VNTRplus database (http://www.miru-vntrplus.org) [27].

### Data entry, management and analysis

The patient Information collected using a structured questionnaire was double entered using Microsoft Access and validated to remove duplicates and data entry inconsistencies. All inconsistencies in the electronic database were resolved by cross checking with the structured questionnaire for clarification. Data was analysed using Stata v.14 (StataCorp, College Station, TX, USA). Statistical comparisons were computed using Chi‑square and Fisher’s exact tests as appropriate. The level of significance was set at p < 0.05.

## Results

### Characteristics of the participants and epidemiological associations

The characteristics of the study participants are reported in Table 1. A total of 2990 smear positive TB patients consented to participate in the study. The majority, 70.5%; (2107/2990 were males with a median age of 40 years (range 13–86), and the remaining 29.5% (883/2990) females with a median age of 33 (range 13–82). Two thirds of the study participants; 78.3% (2340/2990 consumed alcohol on a regular basis and 7.5% (226/2990) smoked (Table 1).

**Table 1:** Demographical and clinical characteristics of participants.

Characteristics	HBA1C<6.5% (TBNDM) N = 2708 n (%)	HBA1C≥6.5% (TBDM) N = 282 n (%)	p-value
Gender (2990)			
Male	1907 (70.4)	200 (70.9)	0.891
Female	801 (29.6)	82 (29.1)	
Age^1^ (2901)			
3–24	407 (15.0)	44 (15.6)	0.965
25–34	635 (23.4)	64 (22.7)	
35–44	673(24.9)	70 (24.8)	
45–54	490(18.1)	56 (19.9)	
55–64	227(8.4)	24 (8.5)	
65+	194(7.2)	17 (6.0)	
TB Status^2^ (2967)			
New	2583 (95.4)	256 (90.8)	<0.001
Relapse	98 (3.6)	18 (6.4)	
Defaulter	4(0.1)	8 (2.8)	
Alcohol consumption (2990)			
Yes	2113 (78.0)	227 (80.5)	0.363
No	595 (22.0)	55 (19.5)	
Smoking Status (2990)			
Yes	207 (7.6)	19 (6.7)	0.722
No	2501 (92.4)	263 (93.3)	
Education^3^ (2196)			-
Primary	260 (9.6)	26 (9.2)	0.314
Secondary	1169 (43.2)	135 (47.9)	
Tertiary	196 (7.2)	25 (8.7)	
No Education	334 (12.3)	51 (18.1)	
Smear positivity (2990)			-
Scanty	124(4.6)	20 (7.1)	<0.001
+1	394(14.5)	21 (7.4)	
+2	1414 (52.2)	183 (64.9)	
+3	776 (28.6)	58 (20.6)	
Cavitation^4^ (2832)			-
Lower lung	90 (3.3)	242 (85.8)	<0.001
Upper lung	2483 (91.7)	17 (6.0)	
Clinical Signs			
Cough	2618 (96.6%)	262 (92.9%)	0.003
Fever	2105 (77.7%)	200 (70.9)	0.011
Hemoptysis	2083 (76.9%)	173 (61.3%)	<0.001
Weight loss	1733 (63.9%)	222 (78.7%)	<0.001
Poor appetite	1462 (53.9%)	179(63.5%)	0.002
Night sweat	1170 (43.2%)	153 (54.3%)	<0.001
Dyspnea	1775(65.5%)	149 (52.8%)	<0.001
Chest pain	2018(74.5%)	166 (58.9%)	<0.001
Fatigue	1590(58.7%)	204 (72.3%)	<0.001

^1^ = 89 missed data for Age

^2^ = 23 missed data for TB status

^3^ = 794 missed data for Education

^4^ = 158 missed data for Cavitation

Two hundred and eighty-two out of the 2990 TB patients (9.4%; 200 males and 82 females) were confirmed as diabetic with HbA1c > 6.5%, 5 (0.2%) s pre-diabetic and 2703 (90.4%) as non-diabetic. The proportions of DM among TB patients was approximately twice as much as the proportion in the general Ghanaian population (9.4% vs 4.0%). Two hundred and fifty-six out of 282 (90.8%) of the TBDM patients were categorised as new cases; 18 (6.4%) relapse and 8 (2.8%) defaulted cases (Table 1).

All patients presented with cough with expectoration and fever as their main symptom. Whereas weight loss (p<0.001), poor appetite (p<0.002), night sweat (p<0.001) and fatigue (p<0.001) were observed in significantly higher proportions among TBDM participants, cough (p<0.003), fever (p<0.011), hemoptysis (p<0.001), dyspnea (p<0.001) and chest pain (p<0.001) were observed in significantly higher proportions among TBNDM.

Sputum bacillary load at presentation was significantly higher in the TBDM group (183 (64.9%) patients having sputum grades of 2+ compared to 1414 (52.2%) patients in the TBNDM group, p<0.001. At the end of the intensive phase of therapy, a significant number of TBDM patients 14.9% (42/282) remained smear-positive compared to 3.4% (93/2708) non-diabetic TB patients (p<0.001).

### TB treatment outcome

Anti-TB treatment outcome varied between the two groups; while 85.0% (2301/2708) of non-diabetic TB patients had a successful treatment outcome, only 65.2% (184/282) TBDM patients had successful treatment outcomes (p<0.001). The proportion of death of 6.4% (18/282) observed in the TBDM patient group was higher compared to 1.1% (30/2708) deaths seen in the non-diabetic TB patients (p = 0.002) (Table 2). All examined TB cases had localised abnormal chest X-ray except for one showing severe cases of infiltration and presence of macro nodules in all six zones of the chest coupled with cavitation at the right upper zone. Among TBDM patients with involvement on CXR, 85.8% (242/282) had isolated lower lung field involvement and 17 (6.0%) patients had upper zone involvement. This is in contrast with lung involvement seen among the non-diabetic TB patients, where 91.7% (2483 /2708) had upper zone involvement.

**Table 2:** Treatment outcome among participants.

Outcome	TBNDM (%) HBA_1C_<6.5% N = 2708	TBDM (%) HBA_1C_≥6.5% N = 282	p-value
Cured	2301 (85.0%)	184 (65.2%)	<0.001
Failure	102 (3.8%)	63 (22.3%)	<0.001
Died	30 (1.1%)	18 (6.4%)	<0.001
Completed	275 (10.1%)	17 (6.0%)	0.026

### Association between MTBC lineages and DM status

Based on SNP typing, we identified six out of the seven human-associated MTBC lineages (Lineages 1, 2, 3, 4, 5 and 6) among TBDM study population. We obtained TB lineage data for 77% (2304/2990) of the total participants comprising 79.7% (1836/2304) Mtbss and 20.3% (468/2304) Maf. Among the TBDM group, we obtained TB lineages for 229 out of 282 isolates: Lineage1-4, 176 (76.9%), Lineage 5, 29 (12.7%) and Lineage 6, 24 (10.4%). Table 3 compares the distribution of *M*. *africanum* among diabetic TB patients (TBDM) and non-diabetic TB patients (TBNDM). Comparing the infecting TB species, we found that, the burden of TBDM cases was not significantly different (Mtbss; 176, 9.6% and Maf; 53, 11.3%, p = 0.2612). However, in a separate analysis stratified by infecting TB-lineage, we found that compared to Mtbss L1-4 there is some suggestive reason to associate L6 to TBDM (odds ratio (OR) = 1.52; 95% confidence interval (CI): 0.92–2.42, p = 0.072, Table 3).

**Table 3:** Distribution of *Mycobacterium africanum* (Maf) among TBDM and TBNDM Patients.

	TBDM	TBNDM	Total	OR	CI	p-value
**Mtbss**	176 (9.6%)	1660 (90.4%)	1836	1.2	0.85–1.26	0.261
**Maf**	53 (11.3%)	415 (88.7%)	468			
**Mtbss L1—L4**	176 (9.6%)	1660 (90.4%)	1836	1.03	0.65–1.57	0.895
**Maf L5**	29 (9.8%)	266 (90.2%)	295			
**Mtbss L1—L4**	176 (9.6%)	1660 (90.4%)	1836	1.52	0.92–2.42	0.072
**Maf L6**	24 (13.9%)	149 (86.1%)	173			

## Discussion

The main findings of this study were 1) 9.4% of 2990 adult TB patients studied had DM, two-fold higher than the general population average of 4.0%, 2) TBDM patients were associated with weight loss, poor appetite, night sweat, fatigue, death and treatment failures and 3) TBDM patients were suggestively likely to be infected with Lineage 6.

In Africa, information on TBDM comorbidity is limited and the few studies performed to date have shown that increasing DM prevalence leads to a significant increase in TB. Over the last 10 years, Ghana has seen an increase in DM prevalence in the general population from 1.6 to over 4% [22]. This increase is paralleled with the recent results from national TB prevalence survey, which showed a higher prevalence of TB in Ghana; 3 times than earlier reported [28]. The increased numbers could however, be due to the implementation of better diagnostic methods and not necessarily increase in the actual proportion of TB patients in the population. Our study prevalence of 9.4% DM among TB patients is approximately twice as high as in the general population [22] which agrees with the few studies conducted in sub-Saharan Africa that found varying rates of DM among TB patients from 1.9% to 35% [29]. In Tanzania, the prevalence of DM among TB patients was 16.7% compared to 9.4% in a group of matched controls [30]. In a similar study in China, where all TB patients were tested for DM, 6.7% of TB patients had DM compared to 4.3% in the community [31], while in Indonesia 13.2% of TB patients had DM compared to 3.2% in the community [32]. These observations highlight obvious interaction of TB and DM. One reason for this could be a bi- directional reciprocal metabolic factors that affect the two diseases. TB induces glucose intolerance and worsens glycaemic control in people with diabetes whereas DM invariably compromises the body’s immune system which potentially leads to succumbing to TB infection. Our finding of a positive relationship between DM and TB thence highlights the need to screening TB patients for DM and vice versa, particularly in Africa where there is lack of data on the occurrence of DM in TB. Early diagnosis of diabetes and management will be important in ensuring effective management of TBDM patients for a favorable outcome.

There was a significant difference in treatment outcome between TBDM and TBNDM patients. Time to sputum culture conversion is a strong predictor of treatment outcome. Our finding compares with other studies which found bacteriological conversion after 2 to be slower in patients with DM in comparison to those of non-diabetic patients [33–37].

Patients in the TBDM group were also four times more likely to die compared to patients in the TBNDM category. Similar findings have been reported from Portugal [38], Maryland [39], and Malaysia [40]. One plausible explanation may be a higher risk of a DM patient with impaired immunity, because of uncontrolled chronic hyperglycemia, which may interfere with effective eradication of the *Mycobacteria*. Another possible explanation is that, DM may decrease the plasma concentrations of TB drugs, which has been associated with response to TB drugs [41]. This observation calls for in-depth follow-up with pharmacokinetics and immunology studies to consider drug-drug interactions and bioavailable levels of anti-TB drugs as well as immune response during TB therapy among TBDM patients.

Like HIV, DM also compromises the host immune system by effecting macrophage and lymphocyte function, essential for the control of TB thus giving opportunity for increase in prevalence of low virulence genotypes like Lineage 6. This view is supported in our study as well as previous work by others showing an association between Lineage 6 and comorbidities e.g. HIV. Our findings support the opportunistic nature of Lineage 6 disease. Moreover, de Jong *et al*. reported that Lineage 6 was significantly more frequent in elderly TB patients in the Gambia, consistent with both slower progression to active disease [42] and the role of age-related immunosuppression [43]. So far, the role of DM in driving Lineage 6 remains unclear and more studies are needed not only on occurrence of the two diseases but effect on treatment outcome.

The limitation of this study is that the initial random blood glucose was based on glucose measured by a point of care machine, with the potential inaccuracies; however the confirmation by glycated sugar measurement reduced the testing inaccuracy. The strength of this study is that we enrolled large number of study participants from both urban and rural residents and this is the first TBDM study conducted in Ghana. Thus, the data will serve as a baseline for future in-depth studies. A major limitation is our inability to follow patients through the treatment phase to monitor the performance of the glucose during treatment.

Our findings highlight the importance of DM in sub Saharan Africa and hence call for the need to raise awareness on routine screening of TB patients for DM to ensure the use of appropriate treatment regimen.

## Supporting information

S1 Checklist STROBE checklist. (DOC)
